# Case of Foreign Body in the Uterus

**Published:** 1864-08

**Authors:** John G. Meachem

**Affiliations:** Racine, Wis.


### CASE OF FOREIGN BODY IN THE UTERUS.

By JOHN G. MEACHEM, M. D., of Racine, Wis.

A few weeks since I was called to visit a Mrs. W—, who gave me the following history of her case: About a year and a half ago she was treated by a physician for ulceration of the os uteri. She had suffered considerably during the flow of her menses, and had a large leucorrheal discharge during the interval. Solid nitrate of silver had been repeatedly resorted to, for the purpose of cauterizing the ulcerated surface, and after a time it was discovered that the mouth of the womb was too small. Whether it was so in the beginning, or whether it was made so by the repeated applications of the caustic, she could not tell, she only knew that her physician did not observe it until he had treated her a long time.

After the discovery was made he resorted to bougies of Ulmus Fulva for the purpose of dilatation. One bougie after another, increasing constantly in size, had been introduced, until the os was pronounced natural.

My grst visit was made just six months after the last visit of her former physician. The patient informed me that she had suffered much more since her treatment than before, and she was quite sure that it had proved an injury instead of a benefit to her. She had experienced intense pain in the back, through the hips and down the limbs. She had painful micturition, slight chills, and fever daily. A profuse purulent discharge from the vagina, increased by hypogastric pressure. The pain attending the flow of her menses she thought equal to that of parturition. Her appetite was gone, and she was considerably emaciated, though a year before she was quite full and fleshy.

I suggested the use of the speculum as the only means of ascertaining her true condition, to which, however, she demurred, saying that she had no "farther confidence in that instrument," and wished me to resort to some other mode of diagnosis if I could. My firmness at last overcame her objection, and with the utmost difficulty, on account of the soreness and irritability of the parts, I introduced a four-valve speculum. The whole neck of the uterus presented a mass of ulceration. A little firm pressure over the hypogastric region, as before stated, would cause a free flow of pus from the uterine cavity. On introducing the sound the cavity was found to be more than twice its usual size, and by gently manipulating with the instrument a hard, moveable substance could be distinctly felt. The sound was withdrawn, and a pair of long uterine forceps introduced, and search made for the substance indicated. After a few moments a piece of wood, two inches and a half in length and half an inch in breadth, was withdrawn. It was black, and very tough, resembling a piece of whalebone, but upon close inspection proved to be one of the slippery elm bougies used more than six months before. It is needless to say that my patient made a very rapid recovery under simple treatment; the cause of all her late suffering having been this "sliver" in the womb.

Remarks on this case would seem to be needless, as the lesson taught by it is so plain that "he who runs may read."

